# New Elements To Consider When Modeling the Hazards Associated with Botulinum Neurotoxin in Food

**DOI:** 10.1128/JB.00630-15

**Published:** 2015-12-29

**Authors:** Adaoha E. C. Ihekwaba, Ivan Mura, Pradeep K. Malakar, John Walshaw, Michael W. Peck, G. C. Barker

**Affiliations:** a Gut Health and Food Safety, Institute of Food Research, Norwich Research Park, Colney, Norwich, United Kingdom; b Faculty of Engineering, EAN University, Bogotá, Colombia

## Abstract

Botulinum neurotoxins (BoNTs) produced by the anaerobic bacterium Clostridium botulinum are the most potent biological substances known to mankind. BoNTs are the agents responsible for botulism, a rare condition affecting the neuromuscular junction and causing a spectrum of diseases ranging from mild cranial nerve palsies to acute respiratory failure and death. BoNTs are a potential biowarfare threat and a public health hazard, since outbreaks of foodborne botulism are caused by the ingestion of preformed BoNTs in food. Currently, mathematical models relating to the hazards associated with C. botulinum, which are largely empirical, make major contributions to botulinum risk assessment. Evaluated using statistical techniques, these models simulate the response of the bacterium to environmental conditions. Though empirical models have been successfully incorporated into risk assessments to support food safety decision making, this process includes significant uncertainties so that relevant decision making is frequently conservative and inflexible. Progression involves encoding into the models cellular processes at a molecular level, especially the details of the genetic and molecular machinery. This addition drives the connection between biological mechanisms and botulism risk assessment and hazard management strategies. This review brings together elements currently described in the literature that will be useful in building quantitative models of C. botulinum neurotoxin production. Subsequently, it outlines how the established form of modeling could be extended to include these new elements. Ultimately, this can offer further contributions to risk assessments to support food safety decision making.

## INTRODUCTION

The spore-forming Gram-positive anaerobic bacterium Clostridium botulinum and two other clostridia (C. baratii and C. butyricum) commonly found in soil or water environments produce botulinum neurotoxins (BoNTs) (1–3). BoNTs, highly potent substances with an estimated human lethal dose of ∼30 to 100 ng (4, 5), are the most powerful toxins affecting human and animal health. Estimates of the lethality of BoNTs are based on animal experiments and, in a few cases, on the estimated amount of toxin consumed in cases of human foodborne botulism (5) (Table 1). The first description of toxicity was made by Justinus Kerner in Germany in 1793 following the consumption of blood sausage prepared from pork (6). BoNTs are zinc metalloproteases that block neurotransmission in cholinergic nerves by cleaving specific sites of SNARE (soluble *N*-ethylmaleimide-sensitive factor attachment protein receptor) proteins (7). SNAREs are a highly conserved set of proteins involved in the fusing of synaptic vesicles to the plasma membrane, mediating most or possibly all cellular membrane fusion events (8). Cleavage of SNAREs causes muscular paralysis in humans and animals, a condition termed botulism (3, 6, 9). Foodborne botulism presently has a high fatality rate, ∼5 to 10% of cases, and the severity of the disease and the widespread presence and persistence of C. botulinum bacteria make botulism a global health concern and a cause for vigilance (5).

**TABLE 1:** Characteristics of the six physiologically and phylogenetically distinct clostridia that form the BoNT^*a*^

Characteristic	Result for indicated neurotoxigenic clostridium					
	Proteolytic C. botulinum (group I)	Nonproteolytic C. botulinum (group II)	C. botulinum group III	C. argentinense (C. botulinum group IV)	C. baratii	C. butyricum
Neurotoxin(s) formed	A, B, F	B, E, F	C, D	G	F	E
Neurotoxin gene location	Chromosome/plasmid	Chromosome/plasmid	Bacteriophage	Plasmid (?)		
Nonneurotoxigenic equivalent clostridium	C. sporogenes	No species name given	C. novyi	C. subterminale	All typical C. baratii strains	All typical C. butyricum strains
Growth temp (°C)						
Minimum	10–12	2.5–3.0	15		10–15	12
Optimum	37–42	25–30	40	37	30–45	30–37
Minimum pH for growth	4.6	5.0	5.1			4.8
NaCl concn (%) preventing growth	10	5		6.5		
Minimum water activity for growth^*b*^	0.94/0.93	0.97/0.94				
Spore heat resistance^*c*^	0.21 (121)	2.4/231 (82)	0.94 (104)	1.1 (104)		<0.1 (100)
Fermentation of:						
Glucose	+	+	+	−	+	+
Fructose	+/−	+	+/−	−	+	+
Maltose	+/−	+	+/−	−	+	+
Mannose	−	+	+	−	+	+
Sucrose	−	+	−	−	+	+
Trehalose	−	+	−	−	−	+

a Updated from reference 3.

b The values shown are for the humectants NaCl-glycerol.

c Each value is the decimal reduction time in minutes (temperature in degrees Celsius).

Six phylogenetically distinct clostridia (C. botulinum groups I to IV and some strains of C. baratii and C. butyricum) produce seven serotypically distinct BoNTs (serotypes A to G) and more than 40 different subtypes (3, 10). These neurotoxins are responsible for botulism in humans and a range of other mammals and birds (11, 12). While BoNT is highly toxic, it is also an efficient therapeutic tool used as therapy for treating neurological disorders (13, 14).

Considering the physiological differences among clostridia and the highly potent nature of the neurotoxin, limiting the proliferation of C. botulinum strains and their neurotoxin production in food is a major issue in the food-processing industry. One recent development is modified-atmosphere packaging, which strongly inhibits aerobic bacterial growth but has only limited effects on anaerobic bacteria (14, 15). Therefore, the details of the genetic and molecular machinery that drives the synthesis and release of BoNT are of absolute relevance for improving botulism risk assessment and hazard management strategies.

Given the potential risks, standard food safety procedures have been making use of predictive models of C. botulinum growth and toxin production to support decision making. Established mathematical models, which relate to the hazards associated with C. botulinum, are largely empirical. Models describe beliefs concerning the unknown concentrations of C. botulinum spores in the environment, the uncertain inactivation kinetics of populations of spores at high temperatures, and the germination and growth of C. botulinum populations under a variety of physicochemical conditions (16). A recent survey (17) identified several hundred measurements of parameters that describe the inactivation kinetics of C. botulinum group II spore populations during isothermal heating. Several parameterized models, such as those of Whiting and Call (18) and Whiting and Oriente (19), are based on hundreds of laboratory observations and quantify the germination and growth dynamics, or the probability of growth, of C. botulinum cell populations under typical food conditions. Other researchers have generated kinetic growth models (20) and time-to-growth models (21–24). These models have been incorporated successfully into risk assessment processes to support food safety decision making, but these processes include significant uncertainties so that the decision making is often conservative and inflexible. As part of a precautionary approach, most current risk assessments allow for toxin production under any conditions after an exceptionally short time, although it is possible that some minimum criteria exist. In general, the current modeling approach, which has made major contributions to established food safety, does not include genetic information beyond the group level and does not identify elements of regulatory control that are the key to transferability and cell-cell variations (in many situations, foodborne botulism may be driven by very few cells, so that cell variability is a crucial unknown). Current modeling integrates many component processes, such as signaling, permeability, and enzymatic activity, so that opportunities for improved understanding are obscured.

Refinement of the models could be achieved by including within them the details of cellular processes at a molecular level. The complexity of these processes ensures that network models are the best way to encapsulate dependent information. Improved modeling may simultaneously address other outstanding questions concerning the survival strategy of anaerobic organisms and the reasons for more than 40 different BoNT subtypes that vary in potency and duration of action. So far, very few mathematical modeling studies exist in the literature for related organisms at the molecular level. A model describing the role of TcdC—an anti-sigma factor transmembrane protein that destabilizes TcdR (an alternative sigma factor that positively regulates toxin production through interactions with RNA polymerase)—in C. difficile toxin gene regulation networks was published recently by Jabbari and colleagues (25). Other models of the effect of pH-induced gene regulation on solvent production by C. acetobutylicum in continuous culture (26) and the impact of interactions between the Agr quorum-sensing system and sporulation initiation network on the number of spores formed by C. acetobutylicum (27) have also been reported. However, no models at the molecular level exist in relation to C. botulinum.

While the structures and mechanisms of action of BoNTs are reasonably well known, C. botulinum regulation, for BoNT production or for the neurotoxin gene (*bont*), is not fully understood. It is assumed that the quantity of BoNT production is strain dependent and influenced by culture conditions and by the nutritional status of the medium (e.g., nitrogen sources), but the precise mechanisms are unknown. Furthermore, the environmental signals that affect the regulation of the toxin gene (and other associated genes) and that trigger the synthesis of BoNTs largely remain to be elucidated. Several *in vitro* methods have been developed and applied to the monitoring of *bont* gene expression in C. botulinum, including a gene reporter system, competitive reverse transcription (RT)-PCR, and quantitative RT-PCR (28). Experiments indicate a peak in neurotoxin gene expression during late exponential or early stationary phase for C. botulinum group I type A (29, 30) and C. botulinum group II type E (28, 30). However, these studies examined very few time points during population growth so that the full *bont* gene expression profile is not always reported.

Notwithstanding these limitations, the current literature includes several elements that could be instrumental in building mathematical models of BoNT production at the molecular level. Therefore, the aim of this review was to compile what is known about the direct and indirect regulation of toxin production in C. botulinum that could be useful in building quantitative models of BoNT production, as a way of complementing the established form of modeling. This is part of an important current aspiration to include more molecular information in risk assessments of foodborne hazards (31, 32). Models at the molecular level would be “plugged” into current statistical models to obtain more details and flexibility concerning particular parameter values, for instance, the minimum time it takes for toxin to be produced or the dependency linked to the rate of toxin production. Moreover, the use of models that are amenable to simulation and to the analysis of what-if scenarios may permit further formulation of hypotheses on the gene expression profiles and interactions which, after a process of reiterative computer simulation, can guide future experimentation.

## BoNT EXPRESSION, STRUCTURE, AND GENETIC CHARACTERIZATION

**FIG 1:**
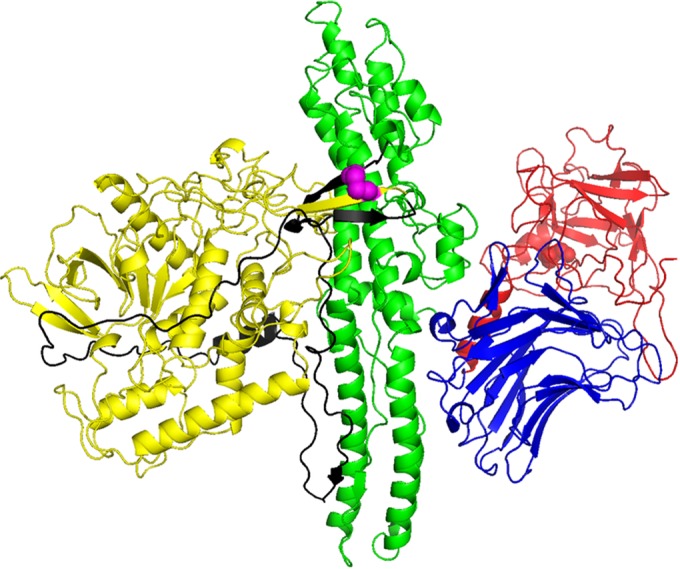
Molecular structure of BoNT type A. Illustrated is the crystal structure of BoNT A1 (BoNT/A1 [Protein Data Bank accession no. 3BTA]), showing the organization of the three functional domains that each play a distinct role in the delivery and action of the toxin. The HC domain, with the receptor-binding C-terminal part in red and the N-terminal part in blue, binds specifically to the nerve terminals; the HN domain, with the translocation domain in green, translocates the L chain (yellow) into the nerve terminal cytosol. The L chain is a metalloprotease that cleaves and inactivates specific SNARE target proteins in order to block chemical messenger release, thus inducing paralysis. The overall structure is 45 by 105 by 130 Å, as reported in reference 33. The image shown was generated with PyMOL script obtained from www.ebi.ac.uk.

*bont* genes are encoded by mobile genetic elements that enable horizontal transfer among different isolates. This is thought to contribute to the evolution of the *bont* loci and thereby to the large number of distinct BoNTs that are currently known. BoNT proteins are initially encoded by a single gene of approximately 3.8 kb and are expressed as a single polypeptide chain (∼150 kDa) (Fig. 1) that is later activated to form a more toxic dichain molecule, by an extracellular bacterial protease (or an added protease such as trypsin). The dichain molecule consists of a heavy (H) chain (100 kDa) and a light (L) chain (50 kDa) held together by a long peptide belt, noncovalent interactions, and a single interchain disulfide bond (shown in black and magenta in Fig. 1 for BoNT/A1) (3, 6). The crystallographic structures of the entire BoNT/A1, BoNT/B1, and BoNT/E1 proteins are known, in addition to some individual domains and L chain-substrate complexes (33–35).

BoNT is released from the bacterium and exists in nature in the form of a complex (36–38), i.e., not as a pure toxin (39). The distinct neurotoxins form complexes of different sizes, ranging from 288 to 900 kDa, by association with nontoxic neurotoxin proteins (ANTPs), i.e., hemagglutinins (HAs) and nontoxic non-HAs (NTNHs), which spontaneously associate with BoNTs at low pH and dissociate at pH ≥7.5. The associated proteins protect the neurotoxin and facilitate its absorption into the body (40, 41).

**FIG 2:**
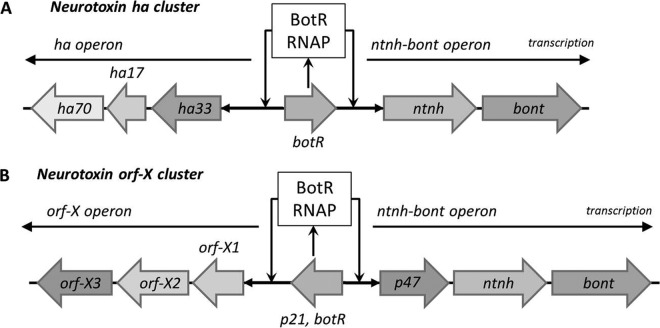
Genetic organization of the neurotoxin gene loci in C. botulinum. This scheme shows the BoNT *ha* (A) and *orfX* (B) gene clusters in serotype A to F strains. For the *ha* cluster, the BoNT and accessory proteins are encoded by two transcriptional units or operons. The first operon includes *bont* and *ntnh*, the second operon encodes three HA proteins (HA70, HA17, and HA33), and each operon is transcribed polycistronically, as indicated by the arrows. The *botR* gene product controls the expression of the genes in the *ha* cluster. In the *orfX* cluster, a *p47* gene is arranged sequentially in the upstream region of the *bont* gene operon and its product is uncharacterized. *botR* is absent from serotype E and some serotype F toxin gene clusters.

The genes encoding BoNTs and ANTPs are located together in two major neurotoxin gene clusters, the “*ha* cluster” and the “*orfX* cluster” (Fig. 2) (9, 42). These clusters are present on either the chromosome or a plasmid in C. botulinum groups I and II, in a bacteriophage in C. botulinum group III, and on a plasmid in C. botulinum group IV. Most strains contain a single neurotoxin gene and one neurotoxin gene cluster, although some strains of C. botulinum group I possess two or three neurotoxin genes and two neurotoxin gene clusters. Recently, strains of C. botulinum group I have been described that contained, unusually, two neurotoxin genes (full type A5 and truncated type B neurotoxin genes) in a single neurotoxin gene cluster (43–45).

For the “*ha* cluster,” the BoNT gene (*bont*) and nontoxic associated genes (*ha* and *ntnh*) are clustered in a locus that contains two transcriptional units (or operons) (Fig. 2A). The first operon (*ntnh-bont*), which is located at the 3′ end of the botulinum locus, encompasses the *bont* gene immediately preceded by the *ntnh* gene. Both genes are cotranscribed in the same orientation, and the organization of this operon is highly conserved in all botulinum toxin-forming clostridia. The second operon contains the *ha* genes and differs slightly among the various subtypes (known subtypes associated with *ha* clusters include neurotoxin genes in subtypes A1, A5, B, C, D, and G). The *ha* operon contains successive genes for the 33-kDa (*ha33*), 17-kDa (*ha17*), and 70-kDa (*ha70*) HAs (1, 46) (Fig. 2A). These *ha* genes are localized upstream of the *ntnh-bont* genes and are transcribed in the opposite orientation (6, 36, 47). The two operons have consensus −10 and −35 core promoter sequences, which is recognizable by a gene encoding a sigma 70 factor (*botR*) that directs RNA polymerase to regulate the genes positively in the neurotoxin gene cluster (48). The *botR* gene encodes a product with features of a DNA-binding protein (i.e., a highly basic isoelectric point and a helix-turn-helix motif) and is localized between the two operons in serotype B strains and some serotype A and F strains and at the 5′ end of the botulinum locus of serotype C and D strains (6). Additionally, *botR* is transcribed in the same orientation as *bont. botR* from serotype A has been characterized as a transcriptional activator of *bont* and *ha* genes on the basis of *botR* overexpression or partial inhibition by antisense mRNA in C. botulinum (1, 48, 49).

The “*orfX* cluster” consists of *bont*, *ntnh*, and sigma 70 factor-encoding (also known as *p21* or *botR*) genes; a group of three open reading frames (*orfX3*, *orfX2*, and *orfX1*); and a single *p47* gene of unknown function (Fig. 2B). *botR* is absent from serotype E and some serotype F toxin gene clusters (6). The three *ha* genes are absent from the *orfX* cluster, while it appears that *bont*, *ntnh*, and *p47* are cotranscribed, as are *orfX1*, *orfX2*, and *orfX3*, from conserved neurotoxin gene cluster promoters (6). The functions of the proteins encoded by *p47*, *orfX1*, *orfX2*, and *orfX3* and their roles (if any) in the neurotoxin complex remain to be established. The known subtypes associated with the *orfX* cluster include type A1, A2, A3, A4, E, and F neurotoxin genes. The subtype A1 neurotoxin gene in single neurotoxin gene strains is more commonly found in the *ha* cluster than the *orfX* cluster, whereas in all dual neurotoxin gene strains where the gene cluster has been sequenced, the subtype A1 neurotoxin gene is in an *orfX* cluster. While *botR* homologs have been identified in *orfX* clusters present in strains of C. botulinum group I, they are absent from strains of C. botulinum group II and C. baratii F7 (50). The sequencing of further genomes will undoubtedly provide more information on neurotoxin gene clusters (9, 10, 36, 44, 46, 51–57).

Quorum sensing has also been implicated in the positive regulation of *bont* gene expression. Cooksley and colleagues (58) provided the first evidence that *agrBD*-dependent quorum sensing regulates BoNT/A production. Furthermore, the transition state regulator CodY, which was previously shown to be an important regulatory link between metabolism and virulence factor synthesis in many low-G+C Gram-positive pathogens (59, 60), was recently suggested as a positive regulator of *bont* gene transcription and BoNT production, as biochemical evidence suggests that CodY interacts with a 30-bp region in the promoter of *bontA* (61). Zhang et al. (61) went further to show that even though inactivation of *codY* did not essentially affect growth, for C. botulinum group I subtype A1 strain ATCC 3502 cells, there was a 50% lower *bont* transcript level than in those of the wild type.

While *bont* gene expression appears to be tightly regulated through positive regulatory elements, including the participation of BotR (49), CodY (61), and an Agr quorum-sensing system (58), negative regulators have also been implicated in BoNT control. The first reported evidence of negative regulation of *bont* synthesis showed that the two-component system CBO0787/CBO0786 (equivalent to CLC_0842/CLC_0843) repressed *bont* synthesis because the CBO0786 response regulator directly binds to the conserved −10 site of the core promoter of *ntnh-bont* and *ha* operons and so blocks BotR-directed transcription (62). Other two-component signal transduction systems (CLC_1093/CLC_1094, CLC_1914/CLC_1913, and CLC_0661/CLC_0663) have also been proposed to regulate *bont* synthesis but only positively (1).

This evidence could support the construction of a signal transduction and sensory transcription regulatory network to describe the kinetics of neurotoxin production.

## MOLECULAR NEUROTOXIN COMPLEX ASSEMBLY PATHWAY

The molecular architecture of the neurotoxin complex is largely unknown, with the exception of structures revealed previously by electron crystallography (30 Å) and electron microscopy (63, 64) and recently by a combination of X-ray crystallography, single-particle EM, and three-dimensional reconstruction (3D-EM) (65). The neurotoxin complexes composed of BoNT and several ANTPs that noncovalently associate with the neurotoxin to form progenitor complexes (PTCs) (40, 41) have been shown by ultracentrifugation to adopt three sizes: 12S (∼288 kDa), 16S (∼500 kDa), and 19S (∼900 kDa) (66, 67). Subtype A1 neurotoxins (BoNT/A1), for example—which so far are the best-characterized neurotoxins, a consequence of both their frequent involvement in human botulism worldwide and their greater potency and therefore suitability for therapeutic use (4)—produce 12S, 16S, and 19S PTCs (the 19S PTCs may represent a dimer of 16S complexes). Their ANTPs include the NTNHA (which, together with BoNT, forms the minimally functional progenitor toxin complex [M-PTC]), and three HA proteins (HA70, HA17, and HA33) that assemble with the M-PTC to form the large-size toxin complex (L-PTC) (8, 41, 65). Type B, C, and D strains produce the 12S and 16S PTCs—i.e., type B to D strains produce M-PTC (BoNT-NTNHA complex) and L-PTC (BoNT-NTNHA-HA complex). The 16S PTCs include BoNT, NTNH, H33, HA17, and HA70, while type A2, E, and F strains do not have the *ha* genes and produce only the 12S PTCs, a noncovalent complex of BoNT and NTNH—i.e., only M-PTC (68) (refer to Table 2 for details).

**TABLE 2:** Clostridium BoNTs and toxin complexes^*a*^

BoNT or toxin complex	Approximate size (kDa)	Component(s)	Toxin type(s)	Reference(s)
Single polypeptide chain	150	BoNT	A, B, C, D, E, F, G	3, 6, 85
M-PTC	288 (12)^*b*^	BoNT, NTNH	A, B, C, D, E, F, G	67, 86, 87
L-PTC	490–760 (16)	BoNT, NTNH, HA(s)	A, B, C, D, G	8, 41, 65, 67–71, 87
LL-PTC^*c*^	900 (19)	Probably a dimer of L	A	63

a Modified from reference 88.

b Each value in parentheses is the sedimentation coefficient in Svedberg units.

c LL, extra-large size.

**FIG 3:**
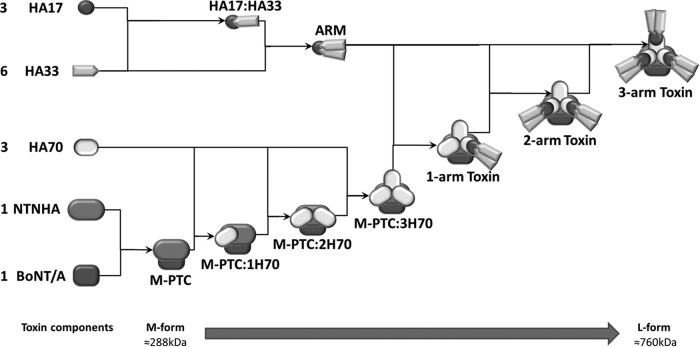
Proposed model of the botulinum toxin complex assembly pathway for subtype A1 neurotoxin. Illustrated is the proposed assembly pathway, showing how the neurotoxin from subtype A1 (BoNT/A1) interacts with its associated proteins (NTNH and HAs) to form the minimally functional progenitor complexes (M-PTCs) and finally the large protein complex (L-PTC). The interaction between BoNT/A1 and NTNHA yields M-PTC. The assembly of M-PTC with three HA70 molecules forms the intermediate M-PTC/HA70 complex, while further conjugation of the M-PTC/HA70 complex with three HA33/HA17 complexes (in a 1:2 ratio depicted as “arms”) leads to the formation of mature L-PTC. The interactions between the components indicated by solid arrows and the stoichiometry numbers (on the left of the diagram) are also shown. Sources for the (possible) masses of M-PTC and L-PTC (shown at the bottom) were all obtained from reference 65 and calculated from ExPASy (84).

Even with these discoveries, there is still limited information regarding the molecular architecture of the 16S and 19S BoNT PTCs. A trigonal symmetry is suggested by individual electron microscopy (EM) micrographs of the BoNT/D 16S PTCs (64). This suggests a structure of the complex that has three extended “arms.” Various structural assembly pathways or models have been proposed for the L-PTC. A report by Bryant et al. (69) suggested a complex composed of BoNT, NTNH, HA70, HA17, and HA33 in a 1:1:2:2:3 ratio for L-PTC/A, whereas earlier studies suggested a stoichiometry of 1:1:3 to 5:5 to 6:8 to 9 (70) or 1:1:3:3:4 (71) for L-PTC/A or 1:1:2:4:4 for L-PTC/D (72). In comparison, electron microscopy (EM) studies on L-PTC/A, L-PTC/B, and L-PTC/D supported a stoichiometry of 1:1:3:3:6 (64, 65, 67). Using a combination of X-ray crystallography, single-particle EM, and three-dimensional reconstruction (3D-EM), Lee et al. (65) found that L-PTC/A consists of two structurally and functionally independent subcomplexes, the M-PTC and the HA complex. The HA complex is composed of HA70, HA17, and HA33 in a 3:3:6 stoichiometry and adopts an extended three-blade architecture, whereas the M-PTC is situated at the top of the HA complex platform. With these results, the assembly pathway for mature L-PTC/A1 formation from individual subcomponents has been proposed to take the following order (Fig. 3) (based on information inferred from references 65 and 68). (i) The association of 1 × BoNT and 1 × NTNHA yields the M-PTC, (ii) the assembly of the M-PTC and 3 × HA70 forms the intermediate M-PTC/HA70, and (iii) further conjugation with the [3(1 × HA17) + (2 × H33)] HA33/HA17 complex leads to the formation of mature L-PTC.

In addition to these toxin complexes, two HA-negative L-PTCs (610 and 680 kDa) found in serotype C and D strains suggest intermediate products in the pathway leading from the 490-kDa M-PTC/HA70 complex to mature 760-kDa L-PTC, which has a smaller number of HA33/HA17 complexes than mature L-PTC (68).

This evidence provides support to develop a structural model of a protein complex that corresponds to neurotoxin transport. Currently, there is insufficient evidence to provide values for important reaction rates.

## CONCLUSIONS AND FUTURE PERSPECTIVES

**FIG 4:**
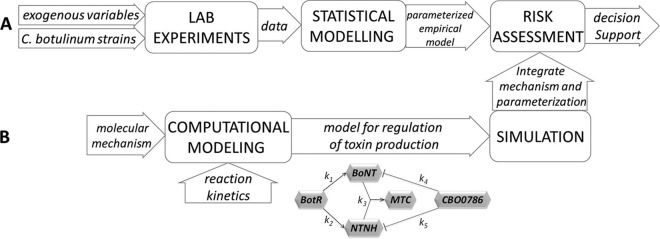
Schematic view of integrated components for risk assessment. The flow diagram shows how the established process for modeling risk can be combined with a systems biology approach to understand a biological mechanism. (A) The established framework for risk assessment. (B) Systems biology approach to biological understanding and integration into risk assessment for C. botulinum toxin production.

On the basis of work previously described in the literature and reviewed here, we have identified existing elements that could be included in the next generation of mathematical models of C. botulinum and hence support advanced risk assessments of botulism hazards. Understanding of the mechanisms that determine the initiation, production, and release of the neurotoxin in more detail not only provides new targets for therapeutic intervention against oral BoNT intoxication but can also guide the development of new mechanistic and quantitative models that would promote opportunities for improved food safety. We envisage a refinement of the current modeling approach whereby the molecular details of the genetic regulation network for toxin production (and the assembly pathway) are built into mechanistic models and incorporated into the risk assessment practices. The flow diagram in Fig. 4A shows the established process of risk assessment, which builds upon sets of experimental data. Established data depend on a range of exogenous variables (such as time, temperature, or the composition of the atmosphere) and on properties of the culture medium (e.g., abundance of nutrients, pH, salt/sugar content, etc.) to define empirical models with predictive power. Figure 4B describes how modeling at the molecular level could support improved molecular data-driven C. botulinum hazard assessment. Using both the qualitative results of experimental work (i.e., the molecular interaction networks) and the quantitative information obtained therein (i.e., the reaction rates), predictive models suitable for simulation and capable of providing predictions can be generated. In addition to the elements identified above, a model of C. botulinum sporulation and germination is also essential for quantifying botulism hazards. While corresponding models of other spore-forming bacteria are well advanced (73–83), for C. botulinum, important elements such as signal transduction remain to be identified. Such understanding would be beneficial in developing new strategies to manage and control botulism and potentially would contribute to improved methods for the production of a toxin for therapeutic use.
